# Spectrum of multi-detector computed tomography imaging findings of thoracic vascular injuries secondary to blunt chest trauma: Correlation with vascular intervention and patient outcomes

**DOI:** 10.4102/sajr.v23i1.1709

**Published:** 2019-07-23

**Authors:** Sithembiso M. Langa, Nondumiso N.M. Dlamini, Balasoobramanien Pillay

**Affiliations:** 1 Department of Radiology, College of Health Sciences, University of KwaZulu-Natal, Durban, South Africa; 2 Department of Vascular /Endovascular Surgery, College of Health Sciences, University of KwaZulu-Natal, Durban, South Africa

**Keywords:** Blunt chest trauma, MDCT findings, vascular injuries, vascular intervention, Durban

## Abstract

**Background:**

Thoracic vascular injuries following blunt chest trauma are the second leading cause of trauma-related deaths. Multi-detector computed tomography (MDCT) is the imaging modality of choice in detecting these injuries.

**Objectives:**

To determine the spectrum of vascular injuries detected on MDCT imaging in patients who sustained blunt chest trauma, and to assess the various types of management options and patient outcomes.

**Method:**

We retrospectively reviewed archived medical records of polytrauma patients who presented with blunt chest trauma and confirmed vascular injury on MDCT and vascular intervention images between May 2015 and August 2018 at Inkosi Albert Luthuli Central Hospital.

**Results:**

Thirty-nine patients with vascular injury findings were analysed. The injury spectrum comprised: 15 aortic injuries (AI), 19 non-aortic injuries (NAI), 4 combined (AI and NAI) and 1 aorto-venous injury. A majority of males (69%) with an overall mean age of 39 years constituted the study cohort. The commonest injury mechanisms included motor vehicle collisions (61%) and pedestrian accidents (28%); the remaining 11% were shared amongst motorbike accidents or falling from a moving train or a height. The subclavian artery (36%) was the most common anatomical location in the NAI and the frequent imaging finding was vessel occlusion (55%). The most common imaging findings in AI were the indirect signs (20.5%) followed by a grade III injury (15.4%). Six patients with a grade III AI were successfully managed with endovascular repair.

**Conclusion:**

A thorough knowledge of blunt vascular injury spectrums and imaging manifestations is critical when interpreting MDCT scans. Awareness of the mechanism of injury will trigger a high index of suspicion and probe a search for a vascular injury.

## Introduction

Trauma-related morbidity-mortality is a worldwide burden. Blunt chest trauma accounts for approximately 20% of the injury mechanisms.^1,2,3,4,5^ In our setting, the prevalence of aetiologic blunt trauma mechanisms of motor vehicle collisions, pedestrian accidents and falling from a height has a shared similarity to published data in first-world countries; however, variation is noted in respect of other compounding injury mechanisms in our setting such as assaults, interpersonal violence and train accidents.^3,6,7^

Imaging plays a pivotal role in patient management, as clinical signs and symptoms in trauma patients may be unreliable because a majority of the patients present with impaired consciousness.^8,9^ Although chest radiographs are usually the first-line screening modality in most trauma centres, they offer limited potential in determining the presence of a vascular injury.^4,9,10^ The current use of focused assessment with sonography for trauma (FAST) may confirm few additional thoracic trauma findings.^1^ However, contrast-enhanced multi-detector computed tomography (MDCT) triumphs as the imaging modality of choice owing to quick acquisition as well as the ability to diagnose life-threatening and occult injuries that are undetectable on radiographs and ultrasound.^2,10^ With improved image quality and spatial resolution on MDCT, it has curtailed the use of the invasive conventional digital subtraction angiography (DSA) as a diagnostic tool to definitively exclude a vascular injury. Angiography is currently reserved for equivocal MDCT findings or endovascular intervention.^11^ The use of full-body computed tomography (CT), also known as Pan CT, has been advocated in the initial assessment of polytrauma patients and has proved to reduce the mortality rate.^12^

There are no South African data that describe MDCT findings of vascular injuries secondary to blunt thoracic trauma in our patient population. The international literature documents a broad and variable spectrum of vascular injuries resulting from blunt chest trauma. Aortic injuries supersede injuries of other major vessels (i.e. aortic arch branches) according to published data. In this study, we describe the vascular injury spectrum (aortic and non-aortic) together with other associated thoracic injuries. The aim of this retrospective study is to describe the MDCT findings of blunt vascular injuries as well as to assess the types of management plan, vascular interventions and relevant patient outcomes.

## Research method and design

The study was performed at Inkosi Albert Luthuli Central hospital (IALCH) which has a tertiary level 1 trauma unit that serves the Durban metropolitan area and the rest of KwaZulu-Natal. All state patients in the province with thoracic vascular injuries requiring surgical intervention, specialised care and trauma intensive care unit (TICU) management are referred to the centre.

Data of all polytrauma patients with blunt chest trauma referred to the radiology department for MDCT scan who fulfilled our inclusion criteria were obtained from the Radiology Information System (RIS) and Picture Archiving and Communication System (PACS). Clinical data for these patients were obtained from the Hospital Information System (Meditec). Data were collected for the period of May 2015 to August 2018. We included patients who were aged 18 years and older with reported vascular injuries. Exclusion criteria applied to patients younger than 18 years, patients with no reported vascular injuries, patients with only unenhanced MDCT scan performed, patients with no records of a documented radiology report on the system, scans with false positive findings and patients imaged at their base hospital. Data were collected relating to patient demographics (age and gender), mechanism of injury, scan times, scan protocols, reported vascular injuries/ findings, associated thoracic non-vascular injuries, vascular interventions and patient outcomes in terms of their survival, discharge or demise.

The aortic injuries were assigned different categories to aid analysis. Perivascular haematoma and vessel wall irregularity were considered as indirect aortic injury signs, whereas direct signs were assigned grade I (intimal flap finding), grade II (dissection, an intramural haematoma or intimal flap of greater than 1 cm length), grade III (pseudoaneurysm) and grade IV (rupture with contrast extravasation). The non-aortic vascular injury signs were classified in terms of intimal flap, vessel calibre reduction, vessel wall irregularity, occlusion, pseudoaneurysm, perivascular haematoma, contrast extravasation, transection and arteriovenous fistula formation. The scans were reported by medical officers, registrars and consultant radiologists. Selected scans with positive reported vascular injuries were reviewed by a consultant radiologist with a five years’ experience and false positive vascular injury findings were excluded.

Scans were performed using a Siemens Somatom Definition AS and Siemens Somatom Definition Flash. The decision of the imaging protocol was based upon the trauma surgeon’s discretion following clinical assessment. Full-body CT angiograms and chest CT angiograms were performed according to the prescribed department imaging protocol. One millimetre reformats were done and images were analysed in 3-Dimension (3D) and Multiplanar Reformats (MPR).

Data analysis was performed using the IBM SPSS statistics Version 25 software. Descriptive analysis, frequencies of demographic data, mechanism of injury, scan protocols, scan time, vascular injury spectrums and thoracic non-vascular injury spectrums were performed. Data were further analysed to depict correlation between injury types and patient outcomes; correlation between vascular injuries and associated thoracic non-vascular injuries; as well as correlation between the types of patient management and patient outcomes.

### Ethical consideration

Ethics approval was obtained from the Biomedical Research Ethics Committee (BREC:BE 343/18), College of Health Sciences, University of KwaZulu-Natal.

## Results

A total of 420 MDCT whole-body and chest scans were performed for blunt chest trauma in polytrauma patients during the study period (May 2015 to August 2018). There was a male predominance of 316 (74.9%). A majority of scans (257 [61%]) were performed outside working hours. Full-body CT angiogram scan was the most frequent imaging protocol request with a 93% frequency.

Thirty-nine patients with positive vascular findings that satisfied our inclusion criteria were selected. A total of 381 patients were excluded on the basis of these criteria: 72 were under 18 years of age; 284 had no reported vascular injuries and 3 had false positive vascular injury findings on review of the scans; 10 had no formal radiology report; 12 only had unenhanced CT scans. The false positive findings included an aortic intercostal branch, aortic wall calcification and brachiocephalic artery wall calcification, which were all misdiagnosed as tiny outpouchings or pseudoaneurysm.

**FIGURE 1:**
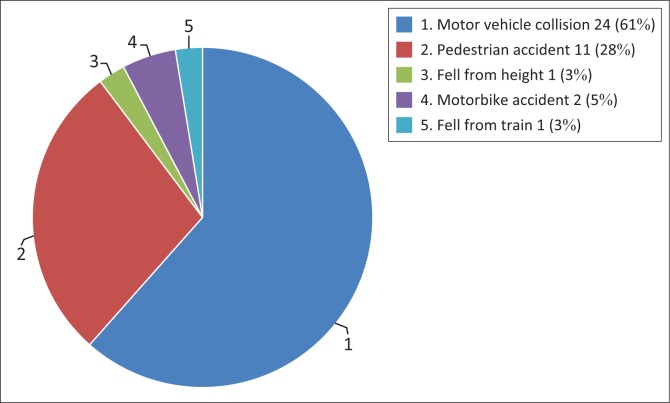
Mechanism of injury – *n* (%).

In the 39 patients with positive vascular findings, males constituted 69% (27/39); with an age range of 20–80 years and a mean of 39.15. Twenty scans were performed outside working hours, and a full-body CT angiogram (see Table 1) was the most requested imaging protocol (31 requests). Figure 1 demonstrates the frequency of recorded injury mechanisms. A total of 15/39 patients had isolated aortic injuries, 19/39 had isolated non-aortic injuries, 4/39 had a combination of aortic plus non-aortic injuries and 1/39 had a combined aortic and azygous vein injury (Table 1). Multiplicity of pathological findings were documented in patients with aortic and non-aortic injury as demonstrated in Tables 2 and 3. In 12.8% of cases, the injuries involved more than one vessel. The most frequent aortic injury imaging findings were indirect signs (20.5%), followed by grade III (15.4%), grade I (10.3%) and grade II (5.1%) findings. There was no record of aortic injury with active contrast extravasation or aortic rupture (grade IV injury). Table 3 depicts a summary of aortic injuries according to the grading system as well as non-aortic injuries. A total of six patients with a grade III aortic injury received endovascular intervention (TEVAR) and were all successfully discharged with 0% mortality reported, (Table 3). One patient that received TEVAR also had a concomitant subclavian artery pseudoaneurysm that was endovascularly stented and involvement of the vertebral artery origin that was coiled. One patient with grade II aortic injury received no intervention because of overall poor prognosis, and another with similar injury was assessed to be stable by the vascular surgery team on discharge and was meant to receive subsequent delayed intervention but was lost to follow up. The remainder of the aortic injury findings of indirect signs and grade I injuries were subjected to conservative management. However, six patients within the aortic injury category inclusive of indirect signs and grade I injury demised as a result of overall poor prognosis related to associated severe extra-thoracic injuries.

**TABLE 1:** Computed tomography scan protocol and types of vascular injury.

Summary of data analysis in selected patients	*n*
**Time of scan**	
During working hours	19
Outside working hours	20
Total	39
**Scan protocol**	
Full-body CT angiogram	31
Chest CT angiogram	8
Total	39
**Vascular injury**	
Aortic	15
Non-aortic	19
Combined aortic and non-aortic injury	4
Combined aortic and venous injury	1
Total	39

CT, computed tomography.

**TABLE 2:** Analysis of computed tomography findings in vascular injury.

Vascular injury spectrum	*n*
**Aortic injuries**	
Intimal flap	9
Dissection: Type B	2
Pseudoaneurysm	6
Wall irregularity	3
Periaortic haematoma	9
**Non-aortic injuries**	
Occlusion	16
Intimal flap	4
Wall irregularity	3
Reduced calibre	3
Perivascular haematoma	2
Pseudoaneurysm	1

Note: Most patients had a combination of imaging findings.

**TABLE 3:** Summary of vascular injuries, injury combinations, frequencies, management and outcomes.

Vascular injury grades	Frequency	%	Management	Outcome		Mortality (%)
				Discharged	Demised	
Aortic grade I	3	7.7	Conservative	1	2	66
Aortic grade I + NAI	1	2.6	Conservative	0	1	100
Aortic grade II	2	5.1	Conservative	1	1	50
Aortic grade III†	5	12.8	Endovascular intervention	5	0	0
Aortic grade III + NAI‡	1	2.6	Endovascular intervention	1	0	0
Aortic indirect signs (venous in one patient)	6	15.4	Conservative	4	2	33
Aortic indirect signs + NAI	2	5.1	Conservative	1	1	50
NAI	19	48.7	Conservative	12	7	36

**Total**	**39**	**100**	**39**	**25**	**14**	**35**

NAI, non-aortic injury.

† , Some patients had co-existing findings of intimal flap and periaortic haematoma.

‡ , One patient with concurrent subclavian artery pseudoaneurysm, vertebral artery origin occlusion and aortic grade III injury (subclavian artery was stented, vertebral coiled and aorta treated with TEVAR).

Note: Indirect signs include periaortic haematoma and/or vessel wall irregularity. Venous injury refers to azygous arch pseudoaneurysm.

**FIGURE 2:**
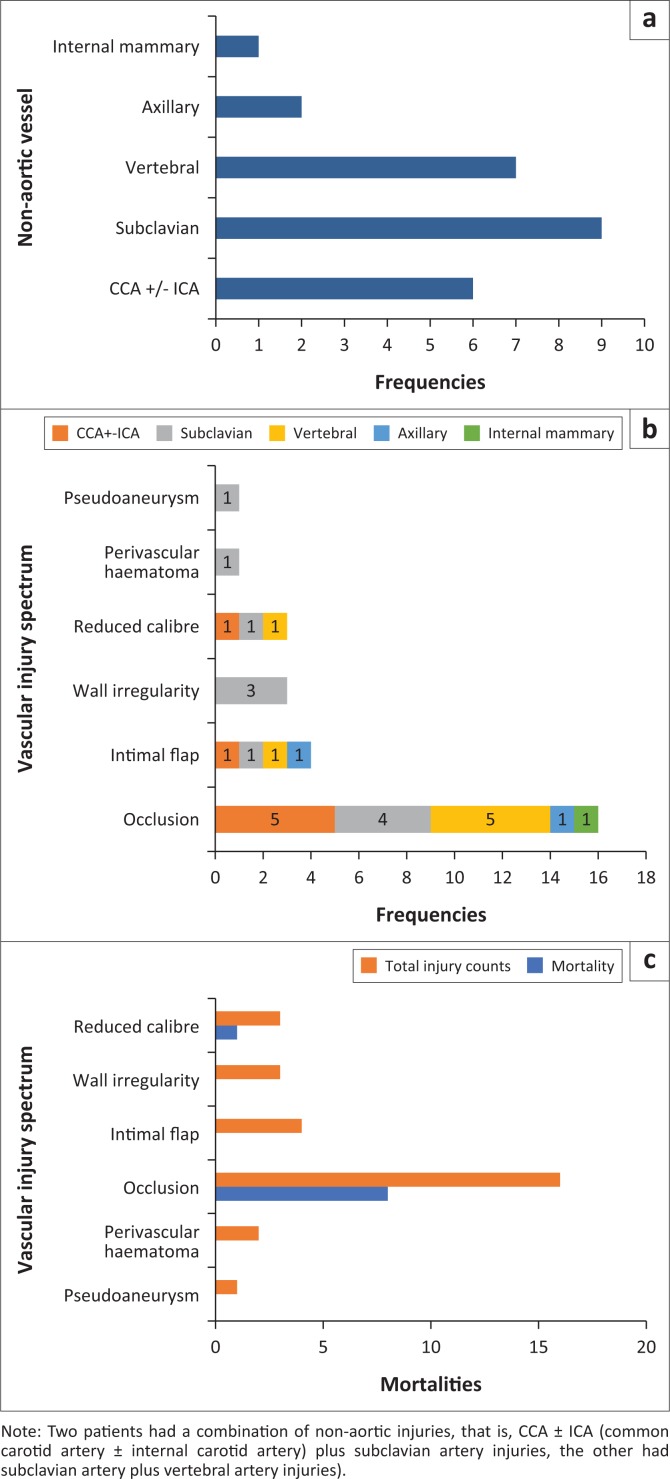
Non-aortic arterial injury (*n*) shows (a) a spectrum of non-aortic arterial vessels; (b) correlates the arterial vessel to injury type; (c) correlates arterial vessel injury type to mortality rate.

**FIGURE 3:**
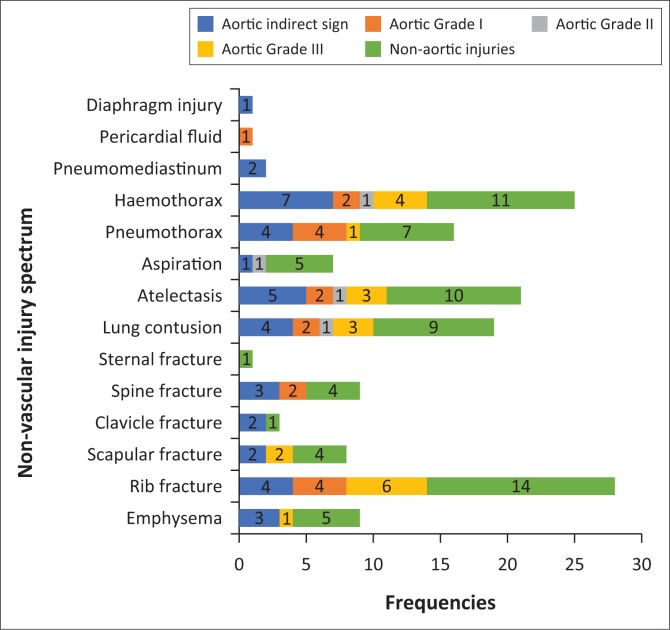
Associated non-vascular thoracic injuries in relation to the vascular injury category.

The vessels involved in non-aortic injuries are demonstrated in Figure 2a, and their respective management is listed in Table 3. The most frequent imaging finding in the non-aortic injuries was vessel occlusion 16/23 (70%), followed by intimal flap 4/23 (17%), wall irregularity and reduced vessel calibre (3/23; 13% each), perivascular haematoma 2/23 (9%) and pseudoaneurysm 1/23 (4%) (Table 2). Figure 2b demonstrates correlation of non-aortic vessels with injury types that were found, and a high incidence of vessel occlusion was seen in carotid and vertebral arteries followed by the subclavian artery. Figure 2c correlates the non-aortic vascular injury types or findings with the mortality rate and a high mortality rate is demonstrated amongst patients with vessel occlusion, particularly with involvement of carotid and vertebral arteries; however, there were other associated severe extra-thoracic injuries. Associated non-vascular thoracic injury findings are represented in Figure 3.

## Discussion

Blunt thoracic vascular injuries following trauma are uncommon, and in our study constituted 9.2% of all injuries in patients referred to the CT department for suspected vascular injury after blunt trauma. Similar to international published data, motor vehicle collision was the most frequent underlying injury mechanism, accounting for 61% in our cases.^5^ Thoracic clinical signs and symptoms may be unreliable in predicting the presence of a blunt vascular injury.^9^ The mechanism whereby there is high velocity and rapid impact or deceleration should raise a high index of suspicion of a blunt vascular injury. These patients usually present with multiple injuries.^4,8,9,13,14^ Other indirect indicators of a vascular injury include a rapid decline in blood pressure with no response to fluid resuscitation and drainage of high volumes of bright red blood from the intercostal drain, usually ≥ 2000 mls.^15,16^ Full-body angiogram MDCT scan protocol is a well-established imaging protocol particularly in polytrauma patient evaluation and has been proved to reduce mortality.^12,17^

At IALCH, a frequently requested full-body protocol entails unenhanced CT brain plus c-spine; post intravenous contrast angiographic phase from base of skull to pelvis ± extremities as determined by the trauma surgeon’s assessment if complex extremity fractures are present; a portal-venous phase abdomen and pelvis; and a delayed phase if there is an indication or suspicion of a renal tract injury. Selective regional chest CT imaging protocol was performed in fewer patients who were fully conscious with pure thoracic blunt trauma or who at least received other extra-thoracic CT scan imaging at the referring base hospital.

**FIGURE 4:**
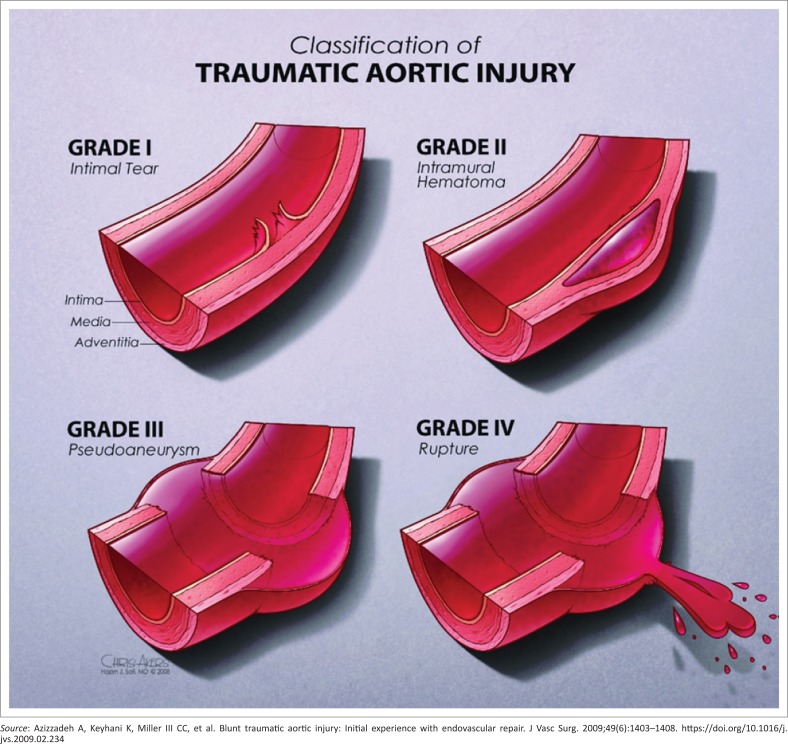
Diagram demonstrating aortic injury classification.

The proposed pathophysiology in blunt vascular trauma is that of a high velocity rapid deceleration process with shearing forces and that of direct blow to a vessel or direct blow to the chest with a rapid increase in intrathoracic pressure or impingement of the aorta between the spine and sternum.^4,12^ The most commonly reported injury profile involving the thoracic aorta typically occurs at the aortic isthmus, which is the junction between the mobile arch and fixed descending aorta, followed by ascending aorta and distal descending aorta.^5,8,9,18,19^ Our study demonstrates a slightly higher incidence of non-aortic injury (23/39) in isolation or in combination with other vessel injuries at 58.9% compared with aortic injury incidence (20/39) (Table 2). Furthermore, multiplicity of vascular injuries in our patients occurred at a rate of 12.8%, which correlates with a report by Gotway and Dawn.^18^ The classification or grading of aortic injuries is demonstrated in Figure 4.^14^ Our study has demonstrated that all grade III aortic injuries occurred at the isthmus (Figure 6). There is a paucity of literature (data) highlighting the non-aortic great vessel injuries when compared with aortic injuries. This is also due to fewer studies that look at all the great vessels, but rather there are case reports and studies looking at individual vessels. Of the non-aortic injuries, the brachiocephalic trunk injuries constitute about 50% of the great vessel injuries, and the other vessels comprise the remainder.^17^ Comparatively we recorded the subclavian artery being the commonest (36%) of non-aortic vessel injuries (Figure 7), followed by the vertebral and carotid artery. The brachiocephalic trunk and common carotid artery injuries tend to occur closer to their origins, whereas subclavian artery injuries typically occur more distally.^4^ The systemic venous and pulmonary circulation injuries are very rare.^4,17^ In our study there was a solitary azygous arch pseudoaneurysm (Figure 8), which occurred concurrently with an aortic indirect injury finding and no intervention was performed owing to other severe extra-thoracic injuries.

**FIGURE 5:**
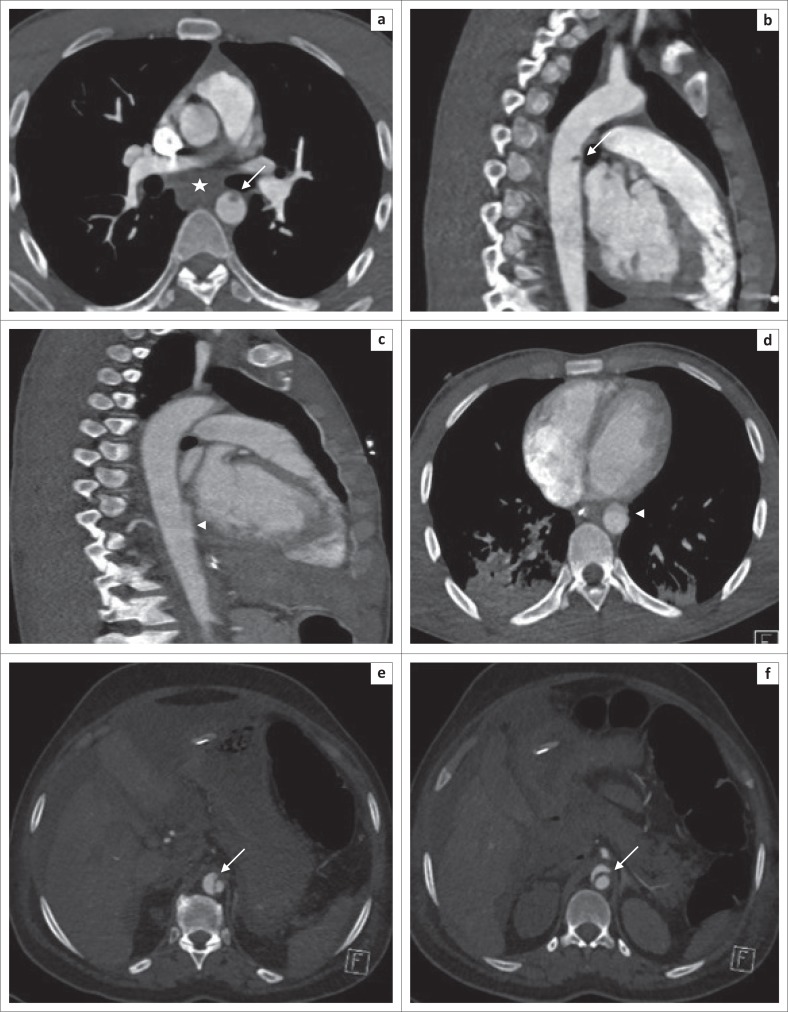
Arrow in image (a) and (b) showing aortic intimal injury (grade I), and a star annotation in (a) is lymphadenopathy not to be confused with mediastinal haematoma. Arrow heads in (c) and (d) demonstrate the lower thoracic aorta anterior wall irregularity (indirect sign) and an elongated intimal flap indicating a focal dissection (grade II). Arrows in another patient in (e) and (f) indicating a more conspicuous aortic dissection (grade II) at the level of the diaphragmatic hiatus.

**FIGURE 6:**
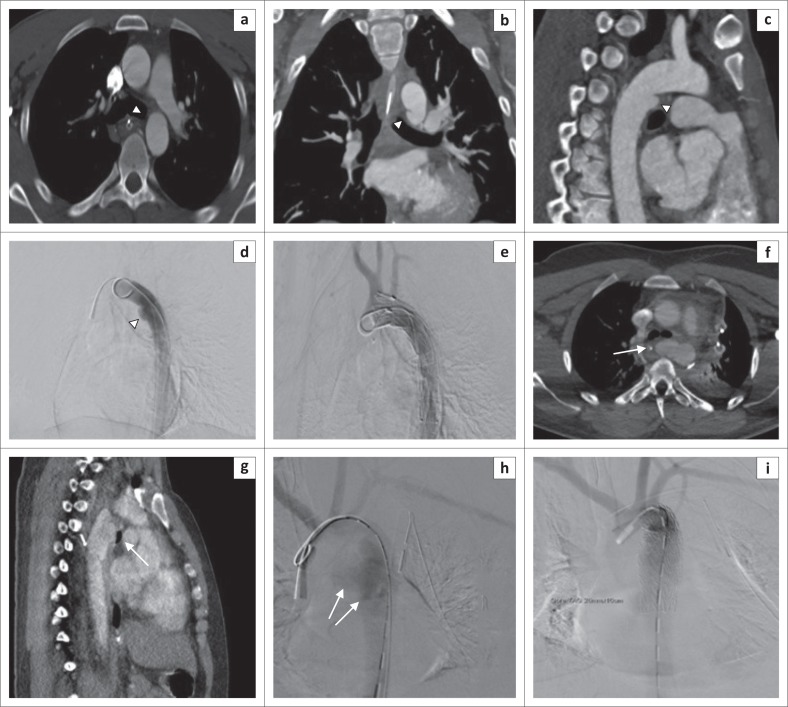
Aortic pseudoaneurysm (grade III) with respective endovascular intervention images in two different patients. Arrow head in (a) shows axial image of a subtle aortic small pseudoaneurysm, which is better demonstrated on MPR formats in (b) and (c). Arrow in another patient (f), (g) shows a larger pseudoaneurysm in the proximal descending aorta and mediastinal haematoma (indirect sign). Subsequent pre-intervention digital subtracted angiograms in (d) and (h) and respective post TEVAR images (e) and (i) demonstrating successful treatment of the pseudoaneurysms.

**FIGURE 7:**
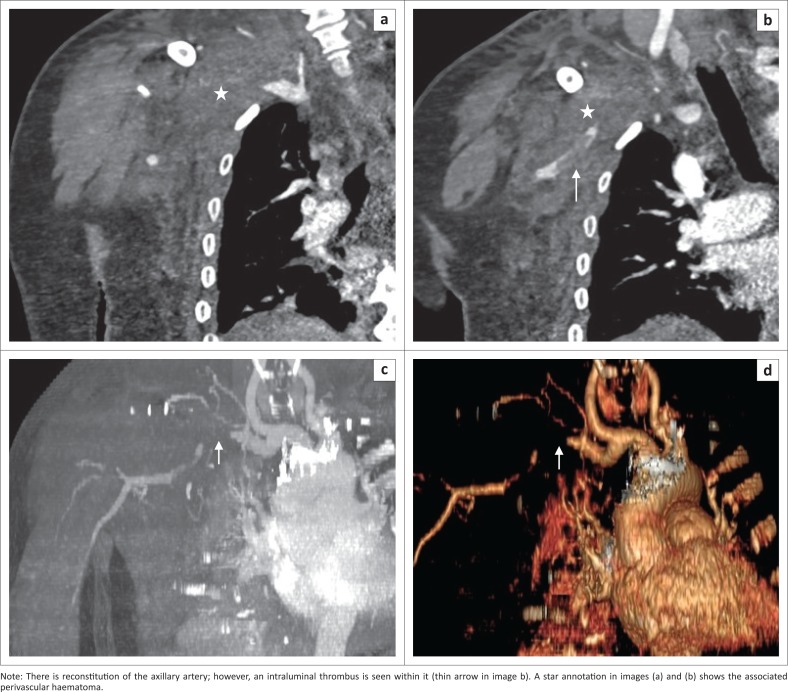
Subclavian artery occlusion and associated haematoma, seen as abrupt cut-off of the right subclavian artery in image (a), better demonstrated in image (c) maximum intensity projection reformat and image (d) 3D volume rendered image, with the arrow.

**FIGURE 8:**
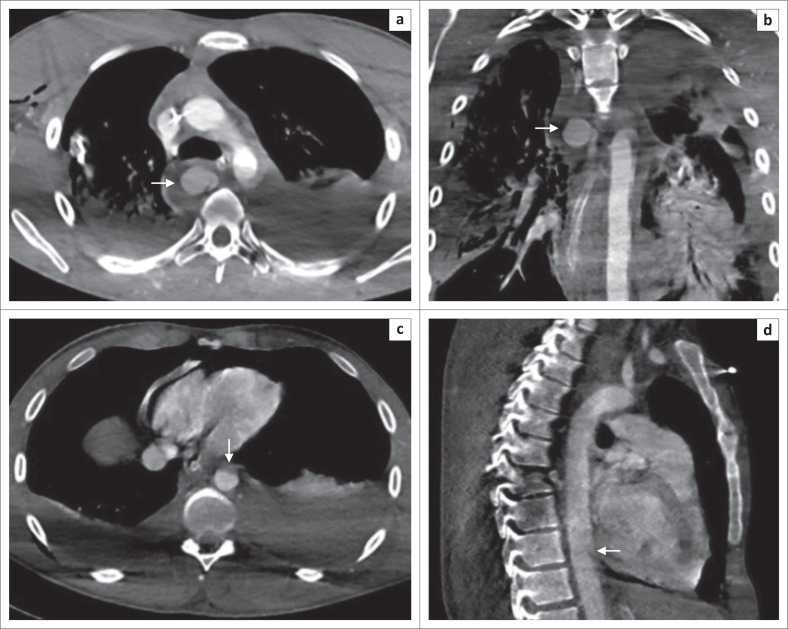
Thick arrows in (a) and (b) demonstrate an azygous arch pseudoaneurysm and surrounding mediastinal haematoma. Arrows in (c) and (d) in the same patient show anterior wall irregularity (indirect sign) in the lower thoracic aorta.

In view of the known high mortality and morbidity related to thoracic vascular injuries, particularly with active bleeding (contrast extravasation), time becomes a crucial factor in detection of these vascular injuries and prompt implementation of definitive management, before serious complications unfold.^4^ MDCT angiogram is the gold standard imaging modality and has shown enormous capabilities in injury diagnosis.^4,5,11,12^ In our setting, the radiology resident trainees, who are almost always the first image interpreters, need to be familiar with the direct and indirect signs of the vascular injury spectrum on MDCT. The use of multiplanar display in image interpretation enhanced by maximum intensity projection (MIP) has been proved to significantly aid the injury detection (Figure 6).^5,11,17^ Blunt aortic injury imaging spectrum is variable and many authors classify the injuries differently.^19^ However, the consistent characterisation is that of indirect aortic injury signs and direct signs. Direct imaging features of aortic vascular injuries include rupture with contrast extravasation (grade IV injury), pseudoaneurysm (grade III injury) which is an outpouching contained within the adventitia, intramural haematoma or dissection (grade II injury) and intimal tear/ flap (grade I injury), which appears as a thin filling defect with or without adherent thrombus (Figures 4, 5 and 6).^14^ Indirect signs include peri-aortic haematoma and wall irregularity or abrupt calibre change.^5,11,12,13,20,21^ We noted the most frequent aortic findings or injury combinations of indirect signs commonly manifesting with periaortic haematomas, followed by grade III injury (pseudoaneurysms), some of which had a co-existing finding of intimal flap in our patients (Table 3). Generally, the grade I aortic injuries or findings of indirect signs are managed conservatively with anticoagulants where necessary, and adequate follow up is important because it is reported that approximately half of the intimal tears of greater than 1 cm will progress to pseudoaneurysms within 8 weeks.^4,9,19^ One of our patients with a grade II injury could not be found for a follow up and another demised. There has been a growing trend with enormous benefits of actively treating the higher aortic injury grades with an endovascular aortic stent graft (also known as thoracic endovascular aneurysm repair [TEVAR]) rather than open surgery which poses physiological challenges and prohibitive costs.^20^

**FIGURE 9:**
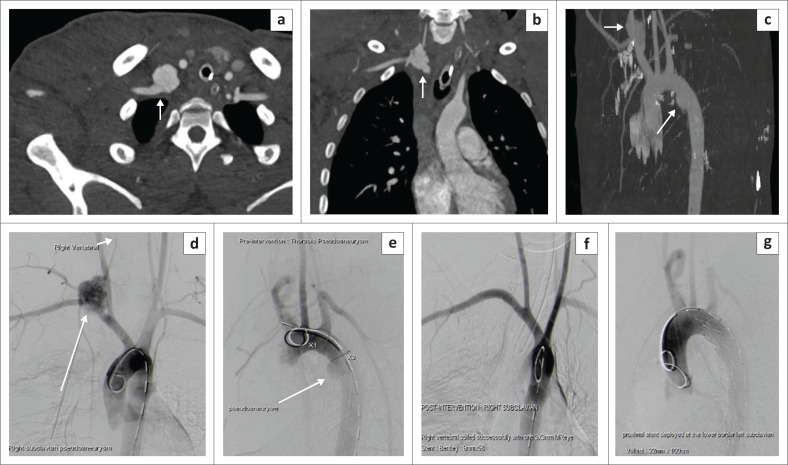
Right subclavian artery pseudoaneurysm. Thick arrows in (a), (b) and (c) demonstrate a large lobulated contained contrast extravasation in keeping with subclavian artery pseudoaneurysm, with a surrounding haematoma. The arrow in (c) MIP reformatted image shows an additional aortic pseudoaneurysm (grade III). Images (d) and (e) are pre-intervention planning images, and these were successfully treated with stenting of the right subclavian artery pseudoaneurysm, coiling of the vertebral artery and stenting of the aortic pseudoaneurysm (f) and (g).

Figure 2 depicts the frequency of non-aortic great vessel arterial injuries in our study, with the subclavian artery being the commonest. The reported blunt vascular injury imaging spectrum of findings in non-aortic injuries includes intimal flap or non-stenotic luminal filling defect, wall thickening reflecting intramural haematoma or dissection, wall irregularity, reduced calibre, focal outpouching or pseudoaneurysm, complete occlusion, transection, arteriovenous fistula formation and an associated perivascular haematoma.^17,22^ Occlusion has been reported to be the commonest blunt trauma manifestation in the common carotid artery.^17^ We also observed complete vessel occlusion to be the most frequent injury in the non-aortic great vessel injury in our patients, and it was also the common finding in all cases with common carotid artery involvement (Figure 2b). The second frequent finding was intimal flap, followed by other less frequent signs such as wall irregularity, reduced calibre and perivascular haematoma (Figure 2b). There was a single count of a pseudoaneurysm with surrounding haematoma which, however, occurred concurrently with an aortic pseudoaneurysm in the same patient, which were all managed with endovascular stenting as per current standard management (Figure 9).

Occlusion of the carotid artery system carries a high morbidity and mortality, and little can be done if there is already established significant brain infarction.^17^ In our study, non-aortic injury, particularly of carotid and vertebral arteries, was observed to carry high mortality (Figure 2b and 2c). However, there was no endovascular or surgical intervention performed, owing to other fatal extra-thoracic injuries.

International literature documents a variety of associated thoracic injuries in patients who sustained blunt vascular trauma. The commonest injury profiles in some patient series included chest wall fractures and pleural based injuries, followed by lung parenchyma injuries (presented as consolidation or contusions or lacerations) occurring moderately, followed by mediastinal injuries and diaphragmatic injuries occuring in lesser frequency than the aforementioned.^1,10^ In our study, we also recorded several associated non-vascular chest injuries or findings, depicted in Figure 3, the commonest being chest wall fractures in both aortic and non-aortic injuries. At face value, it does appear that there is increased likelihood of a vascular injury if there are multiple rib fractures; however, there was no statistical significance established in our study.

## Study limitations and pitfalls

**FIGURE 10:**
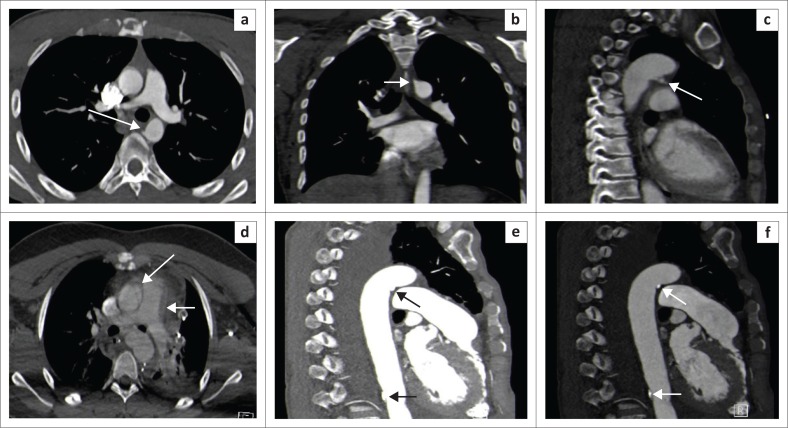
Examples of pitfalls in some of our patients. Long arrows in (a) and (b) demonstrate a thoracic aorta vessel branch mistaken for a small pseudoaneurysm. Image (c) arrow shows an aortic diverticulum with overlying minimal wall calcification. Image (d) arrows show motion artefact, not to be mistaken for dissection. Arrows in image (e) show apparent aortic wall abnormalities mistaken for post traumatic injury, but on adequate adjustment of window settings these were wall calcifications (f).

The study was performed in a single centre and the sample size is small. There was poor patient follow up in both conservatively and endovascularly managed patients owing to discharge home or to referral base hospitals, which may possibly be related to socio-economic factors. The inherent known limitation of artefacts in CT angiography in emergency trauma patients such as motion artefact and poor window settings during image display and interpretation of the study may yield false positive results (Figure 10). In our study, we tried to overcome this by re-evaluating the selected patient images and excluding the patients with false positive findings as per second opinion from a specialist radiologist. We only selected patients with positive vascular findings on CT imaging and therefore false negative results were not assessed. The use of MPR image display can help solve other areas of subtle uncertainty, such as small aortic branches, to avoid misdiagnosis (Figure 10a and 10b). Although cardiac Electrocardiogram (ECG) gated CT angiography is said to minimise motion artefact, it is, however, not a feasible method of examination in an emergency trauma setting. Using appropriate window settings improves the sensitivity of diagnosing subtle findings such as an intimal injury or flap and will eliminate misinterpretation of mural calcifications as vascular injury (Figure 10e and 10f). This can be achieved by the use of a smooth soft tissue reconstruction CT kernel and the use of a wide window setting (approximately 200 level and 800 width) to allow better visualisation of filling defects or wall calcifications from intravascular dense contrast.^23^

## Conclusion

A comprehensive knowledge of the spectrum of MDCT findings for vascular injuries is needed to improve their detection, and a high index of suspicion that is usually based on the mechanism of injury directs a thorough search for vascular injuries. Vascular injuries involved more than one vessel in 12.8% of our cases, alerting radiologists to always search for more than one vessel injury during interpretation of blunt trauma MDCT scans. Most of our patients were treated conservatively and all our grade III aortic injuries were treated with TEVAR with a zero mortality rate, thus indicating a prospect of a promising management outcome with this method of treatment in a small patient cohort.
